# Social Connection and Brain Age in Females and Males

**DOI:** 10.1093/geroni/igaf122.4196

**Published:** 2025-12-31

**Authors:** Helena Blumen, Chava Pollak, Sanish Sathyan, Sofiya Milman, Joe Verghese

**Affiliations:** Stony Brook University, Stony Brook, New York, United States; Stony Brook University, Sto, New York, United States; Stony Brook University, Stony Brook, New York, United States; Albert Einstein College of Medicine, Albert Einstein College of Medicine, New York, United States; Stony Brook University, Stony Brook, New York, United States

## Abstract

Poor social connection is associated with negative health outcomes in aging – including dementia. Structural aspects of social connection include marital status, living arrangements, employment/volunteering, and social networks. Sex-related differences in the structure of social connection have been observed in aging but how they relate to brainageR – or other machine learning estimates of the biological age of a brain – is currently unknown. This study of 171 Ashkenazi Jewish older adults without dementia from the LonGenity study (M Age = 72.59 years; 59% female) aimed to determine if sex modified associations between structural social connection measures (marital status, living arrangements, employment/volunteering, social networks) and brainageR acceleration (AgeAccel). Brain AgeAccel was defined as the residual from regressing machine learning-predicted brainageR estimates on chronological age. Separate linear regression models adjusted for potential confounders were applied to each social connection measure, and the interaction term between sex and social connection was the effect of interest in each model. Living arrangements (alone/with someone) and marital status (married/not married) – but not employment/volunteering (yes/no) or social networks (social network index) – were differentially associated with brain AgeAccel in males and females. Males who lived with someone or were married had lower brain AgeAccel (better) than males who lived alone or were unmarried. Females who lived with someone or were married had higher brain AgeAccel (worse) than females who lived alone or were unmarried. These novel sex-specific associations are interesting but need the be confirmed in larger and more diverse samples of older adults.

